# National Medical Convention

**Published:** 1847-07

**Authors:** 


### THE WESTERN JOURNAL.

New Series.—Vol. VIII.—No. I.

LOUISVILLE, JULY 1, 1847.

### NATIONAL MEDICAL CONVENTION.

In the expectation of receiving at an early day the printed Proceedings of the National Medical Convention lately held in the city of Philadelphia, we give at present only a few additional items, reserving our comments until we shall have before us a full account of the doings of the convention. In another part of this number will be found the report of the committee "on the requirements for the degree of M.D." This report is drawn up with ability, and will be read with interest. The resolutions accompanying it were published in our last number.

The convention having resolved itself into the "American Medical Association," will meet on the first Tuesday of May next, in the city of Baltimore. A report will then be made on the policy of taking from teachers the power to confer degrees. The committee appointed at the first meeting to report on this subject brought in two reports, not having been able to agree in their views. We shall see whether the committee now charged with this duty will be more successful. Few topics, we apprehend, will come before the association better calculated to stir the spirit of controversy. Samson's foxes, it may be, pulled more ways, but they could not have pulled with greater earnestness than our brethren in the association are likely to do, when the question comes up whether teachers shall surrender the licensing power.

On motion of Dr. N. S. Davis, of Binghamton, the following resolution was adopted:

"Resolved, That a committee of one from each State represented in this convention be appointed by the president, whose duty it shall be to investigate the *indigenous medical botany* of our country, paying particular attention to such plants as are now or may be hereafter during the term of their service found to possess valuable medicinal properties, and are not already accurately described in the standard works of our country; and report the same in writing, giving not only the botanical and medical description of each, but also the localities where they may be found, to the next annual meeting of the American Medical Association."

The proceedings of the convention, soon to appear, will include the various reports made during its sitting. So soon as we are in possession of this document, we shall resume the subject. In the mean time, enough has reached us to convince the most skeptical that the labors of this association have not been in vain, and to inspire us with strong hopes for the future. The members who left their business and homes, and incurred the labor and cost of attending these meetings, have entitled themselves to the thanks of the American medical profession.

